# Lactose-Gated Mesoporous Silica Particles for Intestinal Controlled Delivery of Essential Oil Components: An In Vitro and In Vivo Study

**DOI:** 10.3390/pharmaceutics13070982

**Published:** 2021-06-29

**Authors:** Elisa Poyatos-Racionero, Isabel González-Álvarez, Paola Sánchez-Moreno, Leopoldo Sitia, Francesca Gatto, Pier Paolo Pompa, Elena Aznar, Marta González-Álvarez, Ramón Martínez-Máñez, María Dolores Marcos, Andrea Bernardos

**Affiliations:** 1 Instituto Interuniversitario de Investigación de Reconocimiento Molecular y Desarrollo Tecnológico (IDM), Universitat Politècnica de València, Universitat de València, Camino de Vera s/n, 46022 Valencia, Spain; elpora@upvnet.upv.es (E.P.-R.); elazgi@upvnet.upv.es (E.A.); rmaez@qim.upv.es (R.M.-M.); 2 CIBER de Bioingeniería, Biomateriales y Nanomedicina (CIBER-BBN), 46022 Valencia, Spain; 3 Departamento de Ingeniería, Sección de Farmacia y Tecnología Farmacéutica, Universidad Miguel Hernández, 03550 Alicante, Spain; isabel.gonzalez@umh.es (I.G.-Á.); marta.gonzalez@umh.es (M.G.-Á.); 4 Department of Applied Physics, Faculty of Sciences, University of Granada, Avenida Fuente Nueva s/n, 18071 Granada, Spain; paolasm@ugr.es; 5 Nanomedicine Laboratory, Department of Biomedical and Clinical Sciences “Luigi Sacco”, University of Milano, 20157 Milan, Italy; leopoldo.sitia@unimi.it; 6 Nanobiointeractions & Nanodiagnostics, Istituto Italiano di Tecnologia (IIT), Via Morego, 30, 16163 Genova, Italy; francesca.gatto@iit.it (F.G.); pierpaolo.pompa@iit.it (P.P.P.); 7 Unidad Mixta de Investigación en Nanomedicina y Sensores, Instituto de Investigación Sanitaria La Fe, Universitat Politècnica de València, Av Fernando Abril Martorell 106, 46026 Valencia, Spain; 8 Unidad Mixta UPV-CIPF de Investigación en Mecanismos de Enfermedades y Nanomedicina, Centro de Investigación Príncipe Felipe, Universitat Politècnica de València, C/Eduardo Primo Yúfera 3, 46100 Valencia, Spain; 9 Departamento de Química, Universitat Politècnica de València, Camino de Vera s/n, 46022 Valencia, Spain

**Keywords:** essential oil components, mesoporous silica microparticles, controlled release, small intestine, in vitro and in vivo intestinal models, lactase enzyme, molecular gates, enterocyte-like monolayers

## Abstract

Mesoporous silica microparticles functionalized with lactose for the specific release of essential oil components (EOCs) in the small intestine are presented. In vitro and in vivo intestinal models were applied to validate the microparticles (M41-EOC-L), in which the presence of lactase acts as the triggering stimulus for the controlled release of EOCs. Among the different microdevices prepared (containing thymol, eugenol and cinnamaldehyde), the one loaded with cinnamaldehyde showed the most significant Caco-2 cell viability reduction. On the other hand, interaction of the particles with enterocyte-like monolayers showed a reduction of EOCs permeability when protected into the designed microdevices. Then, a microdevice loaded with cinnamaldehyde was applied in the in vivo model of Wistar rat. The results showed a reduction in cinnamaldehyde plasma levels and an increase in its concentration in the lumen of the gastrointestinal tract (GIT). The absence of payload release in the stomach, the progressive release throughout the intestine and the prolonged stay of the payload in the GIT-lumen increased the bioavailability of the encapsulated compound at the site of the desired action. These innovative results, based on the specific intestinal controlled delivery, suggest that the M41-payload-L could be a potential hybrid microdevice for the protection and administration of bioactive molecules in the small intestine and colon.

## 1. Introduction

Hybrid systems based on Mesoporous Silica Particles (MSPs) as inorganic support, coated with molecular gates (also known as gatekeepers or nanovalves), have been widely described and applied in several research fields such as sensing [1,2], chemical communication [3,4,5] or drug controlled release for biomedical or food technology applications [6,7,8,9,10,11,12,13,14,15]. The versatility of these systems lies not only in the use of molecular gates for on-command payload delivery, but also in the great variety of molecules they can harbor in their porous structure. Various examples of molecules encapsulated in MSPs can be found in the literature, including vitamins [16,17,18], antioxidants [19,20], antibiotics [21,22], anti-inflammatories [23,24] or anti-tumor compounds [25,26,27].

Focusing on this field, several examples of MSPs for controlled delivery of bioactive molecules into the gastrointestinal tract (GIT) have been reported [28,29,30,31,32,33]. The multiple conditions present along the digestive system provide a wide variety of stimuli able to trigger the controlled release of the payload lodged into MSPs [30]. Among these stimuli, differences in pH values or redox potential can be highlighted, as well as specific enzymes secreted by the GIT tissues and their sheltered microbiota [34]. In fact, different MSPs have been used to effectively reach the GIT and deliver their cargo through pH-, redox- or enzyme-responsive molecular gates [16,23,35,36,37,38].

From another point of view, one example of bioactive molecules that are gaining great interest is essential oil components (EOCs). Essential oils (EO) are complex mixtures of about 20–60 components, generated by aromatic and medicinal plants in their secondary metabolism, in order to protect themselves against tissue damage (as pests or oxidative stress, among others) [39]. Broadly, EOCs have important nutritional and medicinal properties: antimicrobial [40,41,42], antioxidant [43], anti-inflammatory and anticancer [44]. Due to the aforementioned properties, EOCs have traditionally been used as naturally occurring alternatives to other synthetic chemical compounds in food and pharmaceutical industries as preservatives and antioxidant molecules [45,46]. Their proven anti-inflammatory [43,47,48,49,50,51,52,53,54,55] and/or anti-cancer properties [56,57,58,59], in addition to their natural origin, have been a crucial combination in putting the focus on their study. Furthermore, their weak points as drug molecules [60,61], such as low water solubility due to their lipophilic nature, or their general high volatility, could be mitigated using controlled release systems [12]. Furthermore, their lipophilic nature entails a too-high passive permeability through cell tissues [62,63], which also could be mitigated by the sustained administration provided by controlled release devices. Among the research, some reports describe the use of gated MSPs to deliver EOCs [64]. In fact, the development of smart MSP-systems, loaded with EOCs and functionalized with specific molecular gates, could be a promising combination for enhancing EOC applications in a number of diseases.

To move forwards and validate the applications of gated MSPs loaded with EOCs, it is essential to study their interaction with biological systems and evaluate their possible toxicity by means of in vitro and/or in vivo models. The toxicity of MSP-based materials has been extensively studied in numerous works found in the literature [65,66,67]. Their particle size [68], the presence of organic moieties onto the external surface and their chemical nature [66,69,70,71], the particle concentration and stability [72], the administration route into the organism [73] and the inner toxicity of the host molecules are factors that can modify the toxicity of MSP-systems, from harmless to highly toxic. Focusing on the oral route of administration into the GIT, several useful models to study the particle integrity and their interaction/toxicity with the involved tissues can be found. Progressively in terms of complexity, the in vitro digestion model simulates changes in pH and ionic strength, as well as the presence of biomolecules (including enzymes) in the different organs of the GIT, and has been widely applied [74,75]. Coming up next, more complex in vitro tests, such as GIT cellular models, have also been employed; as, for example, the immortalized Caco-2 cell line of human colon adenocarcinoma. Under certain conditions, this line spontaneously differentiates into enterocyte-like monolayers that closely simulate the epithelium of the small intestine (SI) [76,77,78]. This system, also denominated as the intestinal barrier, is a very appropriate model to mimic and study the interaction of nano- and microparticles with intestinal enterocytes, as well as to obtain an initial understanding of the internalization and permeability processes through the intestinal membrane. Finally, in vivo animal models provide more information on factors that cannot be simulated in the in vitro tests, such as feedback mechanisms, peristaltic movements, gastric emptying or changes in pH and secretion flow rates, as well as intestinal microflora and liver metabolism, which should also be used to study the effect of encapsulated EOCs [74].

Based on the above, we report herein the preparation of EOC-loaded MSPs functionalized with lactose as a molecular gate, which allows EOCs’ on-command delivery in SI conditions (lactase secretion). Despite lactose (Lac) molecules having previously been used as molecular gates [79], its behavior as gatekeeper in MSPs under GIT conditions for delivering EOCs has not been reported yet. The aim of the EOC-loaded lactose-capped MSPs developed in the present work is to increase the bioavailability of EOCs throughout the intestinal lumen. This is expected to be achieved by the combination of different factors that simultaneously occur, such as (i) the increase in the EOCs local concentration thanks to the encapsulation process; (ii) the dependence of the EOCs’ release on the enzymatic activity of the enterocytes-secreted lactase, which makes it a controlled and sustained process over time; and (iii) the reduction of the high EOCs permeability due to progressive administration through the SI, thus favoring a sustained administration and a more prolonged effect throughout the entire intestinal lumen. The system’s effectiveness and the accomplishment of the aforementioned characteristics have been evaluated in this work using in vitro and in vivo biological models in which the secretion of lactase, a β-galactosidase enzyme (β-gal) [80,81,82], acts as trigger stimulus for the hydrolysis of the molecular gate and the payload’s release. The integrity of the system subjected to changes in pH, ionic strength and presence of biomolecules has been studied in an biofluid digestion model, and its GIT-interaction has been assessed with cellular models of Caco-2 and enterocyte-like monolayers (intestinal barriers). The toxicity of the gated particles, internalization, cell viability, inflammatory response and cell permeability are evaluated. Additionally, lactose-gated MSPs loaded with cinnamaldehyde have been tested in an in vivo model of the Wistar rat to evaluate the effectiveness of the microdevice in prolonging the EOC-presence throughout the intestinal lumen. Measurements of the EOC concentration in plasma as well as in the different areas of the GIT have been performed. As far as the authors know, lactase-responsive mesoporous silica microparticles designed for the intestinal controlled release of natural compounds such as EOCs have not been presented before.

## 2. Materials and Methods

### 2.1. Chemicals and Cell-Culture Media

Tetraethylortosilicate (TEOS), triethanolamine (TEAH_3_), sodium hydroxide (NaOH), N-cetyltrimethylammonium bromide (CTAB), (3-aminopropyl)triethoxysilane (APTES), N-(3-dimethylaminopropyl)-N′-ethylcarbodiimide (EDC), lucifer yellow (LY), coumarin 6 (C6), rhodamine B (RhB), lipopolysaccharide (LPS, from Escherichia coli), trifluoroacetic acid (TFA), thymol (Thy), eugenol (Eug), cinnamaldehyde (Cin), lactose (Lac), paraformaldehyde (PAF), bovine serum albumin (BSA), β-galactosidase (β-gal) from Aspergillus oryzae and all the components of the simulated fluids for the biofluid digestion were provided by Sigma (Sigma-Aldrich Química S.L., Madrid, Spain). Ethanol (extra pure), methanol (MeOH) and dimethyl sulfoxide (DMSO) were purchased from Scharlab (Barcelona, Spain). 3-(4,5-dimethylthiazol-2-yl)-2,5-diphenyltetrazolium bromide (MTT) and Hanks’ Balanced Salt solution (HBSS) were supplied by ThermoFisher (Madrid, Spain). Dulbecco’s modified Eagles Medium (DMEM), fetal bovine serum (FBS), 1% nonessential aminoacids and 100 U/mL penicillin/streptomycin were purchased from Labclinics (Labclinics S.A., Barcelona, Spain).

### 2.2. Synthesis of Mesoporous Silica

The synthesis of the mesoporous silica material was performed according to the protocol described in previous authors’ works [16]. The synthesis process was carried out following the “atrane route” [83] in which the molar ratio between the reagents was: 7 TEAH_3_: 2 TEOS: 0.52 CTAB: 0.5 NaOH: 180 H_2_O. In a typical synthesis, NaOH was added to TEAH_3_ and the mixture was heated to 120 °C. Then, the mixture was cooled to 70 °C and 11 mL of TEOS were added dropwise. The reaction was heated under stirring until 120 °C and once the temperature was reached, CTAB was added and the mixture was cooled again to 70 °C. Finally, deionized H_2_O was added, and the mother liquor was stirred vigorously for 1 h at room temperature. The final mixture was transferred to a Teflon flask and kept at 100 °C for 24 h. After this time, the suspension was filtered under vacuum and washed until it reached neutral pH. The material was dried and finally calcined at 550 °C to completely remove the surfactant molecules which conformed the template, thus obtaining the final MCM-41 solid (M41) in the form of microparticles.

### 2.3. Synthesis of the Gated Materials

A fluorophore and three EOCs (Thy, Eug and Cin) were loaded in the M41 microparticles (solids M41-#). RhB was encapsulated into M41 following the immersion method. With that purpose, 1 g of M41 was added to a solution of RhB (0.8 mmol in 40 mL of H_2_O) and stirred for 24 h. The mixture was filtered and the resulting solid (M41-RhB) was dried under vacuum. EOCs (Thy, Eug and Cin) were loaded into the mesoporous material by steam adsorption. For that, 250 mg of M41 was mixed with 250 mg of each EOC in a perfectly closed vial. The mixtures were shaken at 40 °C for 24 h to obtain the loaded solids (named M41-EOC (i.e., M41-Thy, M41-Eug and M41-Cin)). In a second step, the loaded microparticles were reacted with APTES. APTES functionalization was performed by mixing 1.2 g of M41-# with 1.68 mL of APTES in 16 mL of H_2_O for 5.5 h at room temperature. The obtained materials (M41-EOC-N (i.e., M41-Thy-N, M41-Eug-N and M41-Cin-N) and M41-RhB-N, or in general M41-#-N) were collected by centrifugation and dried under vacuum. Finally, the lactose molecule was reacted with the amine groups of the M41-#-N solids following a similar protocol previously described with slight modifications [84]. In a typical synthesis of 2 g of lactose in 40 mL of H_2_O was stirred for 30 min. Then, 1 g of the corresponding M41-#-N solid was added, and the reaction was stirred at room temperature overnight. The final solids were collected by centrifugation and washed three times with deionized H_2_O. Finally, the lactose-functionalized solids (M41-RhB-L and M41-EOC-L (i.e., M41-Thy-N-L, M41-Eug-N-L and M41-Cin-N-L) or M41-#-L in general) were dried under vacuum and stored at 5 °C until their use. Additionally, three EOC-loaded lactose-capped materials with an additional hydrophilic fluorophore (coumarin 6, C6) were synthesized to carry out microscopy experiments. With this aim, C6 was dissolved in the corresponding EOC in melted state (10 mg C6/100 μL EOC) and mixed with 100 mg M41; the loaded solid was reacted with APTES and capped with lactose following a similar procedure to that shown above to obtain the final M41-EOC_C6_-L (i.e., M41-Thy_C6_-N-L, M41-Eug_C6_-N-L and M41-Cin_C6_-N-L) fluorescent materials (also included in the M41-#-L denomination).

### 2.4. Characterization Techniques

X-ray diffractograms were carried out on a Bruker D8 Advance diffractometer (Bruker, Coventry, UK) using Cu Kα radiation. TEM images were obtained with a JEOL JEM-1010 (JEOL Europe SAS, Croissysur-Seine, France). N_2_ adsorption–desorption isotherms were recorded with a Micromeritics TriStar II Plus automated analyzer (Micromeritics Instrument Corporation, Norcross, GA, USA). The samples were degassed at 120 °C in vacuum overnight. The specific surface areas were calculated from the adsorption data in the low-pressure range using the BET model. Pore dimensions (size and volume) were determined following the BJH method. The functionalization process was followed by ζ potential with Zetasizer Nano ZS equipment (Malvern Instruments, Malvern, UK). Samples (bare and functionalized microdevices) were dispersed in deionized H_2_O at neutral pH at a concentration of 1 mg/mL. Before each measurement, samples were sonicated for 2 min to preclude aggregation. Values were calculated from the particle mobility values by applying the Smoluchowski model. Each measurement was performed in triplicate at 25 °C, and the average of three recordings per measurement was reported as ζ potential (error bars correspond to the standard deviation-value). The organic content in loaded and functionalized particles was determined by TGA. Thermogravimetric analyses were carried out on a TGA/SDTA 851e Mettler Toledo balance (Mettler Toledo Inc., Schwarzenbach, Switzerland), using an oxidant atmosphere (air, 80 mL/min) with a heating program consisting of a heating ramp of 10 °C per minute from 25 °C to 1000 °C with three isothermal heating steps of 30 min at 37 °C, 100 °C and 1000 °C. Fluorescence spectroscopy measurements were performed with a JASCO FP-8300 Spectrofluorometer equipped with a FMP-825 microplate reader (JASCO, Easton, PA, USA). UV-Visible spectra were recorder with a JASCO V-650 spectrophotometer. EOCs quantification was performed by HPLC with Novapack C18 column (Waters Alliance^®^ e2695, Milford, MA, USA).

### 2.5. Biofluid Digestion Assay

The employed artificial fluids for the biofluid digestion assays and the residence times in each organ were based on the protocol described by Versantvoort et al. [85]. The pH of the fluids was adjusted to the appropriate value (6.8 ± 0.1 for saliva, 1.3 ± 0.1 for stomach, 8.1 ± 0.1 for duodenal and 8.2 ± 0.1 for bile) with NaOH (1M) or HCl (37%). All the experiment was performed at 37 °C. In a typical experiment, four samples of 10 mg of M41-RhB-L were placed in different vials and the simulated fluids were added in temporary order mimicking the transit of food along the intestinal tract. Each sample was used to reach a subsequent stage of the digestion and samples were taken from each of the phases of the digestion process. Hence, 3 mL of simulated saliva were added to the first sample in order to simulate the mouth stage. The second sample was also suspended in 3 mL of simulated saliva, and after 5 min of incubation, 6 mL of gastric juice were added. Finally, the two remaining samples were treated as the previous one and, to simulate the intestinal stage, after 120 min stirring with gastric juices, 3 mL of bile, 6 mL of duodenal fluid and 1 mL of 1 M NaHCO_3_ were added to both, plus 5 mg/mL of β-gal to only one of them, and these mixtures were shaken until completing the theoretical time of intestinal stay (5 h). The aliquots taken from each digestion-step were separated by centrifugation and the released RhB from the pore voids of M41-RhB-L to the supernatant was determined by means of its fluorescence signal (λ_exc_ = 555 nm, λ_em_ = 572 nm). The pellets were collected for further characterization. In order to remove the major part of the salts and proteins present in the simulated fluids, the M41-RhB-L particles in each sample were collected by centrifugation, separated from the supernatant, washed with deionized H_2_O until neutral pH and dried under vacuum.

For the observation of the fluorescence-confinement of the RhB into the M41-RhB-L during the biofluid digestion process, different aliquots were taken directly from each biofluid digestion fluid without β-gal. 5 μL of each digestion step was taken and dropped onto glass coverslips, and confocal laser scanning microscopy (CLSM) images were acquired in a Leica TCS-SP5 confocal microscope with an oil-immersion 63x objective, and excitation wavelength λ_ex_ = 546 nm. Additionally, images of a suspension of 1 mg/mL of M41-RhB-L in deionized H_2_O at neutral pH were taken as control.

### 2.6. Cargo Release Experiments

To perform the payload release experiments from M41-EOC-L materials (M41-Thy-L, M41-Eug-L and M41-Cin-L), a batch mode experiment was carried out. Hence, 14 samples of 2 mg for each M41-EOC-L were placed in microtubes, half of them suspended in 1 mL of PBS (pH 7.4), and the other half in 1 mL of β-gal solution in PBS (5 mg/mL), and were kept under stirring at 37 °C. At scheduled times (2 min, 30 min, 1 h, 3 h, 6 h, 9 h and 24 h), aliquots were taken and centrifuged in order to remove the suspended microparticles. Proteins present in the supernatants were precipitated with MeOH (1 mL) at −14 °C, and the mixture was centrifuged. EOC delivery from M41-EOC-L to the supernatant solution was analyzed by HPLC (see below).

### 2.7. EOCs Quantification

The EOC concentration in the aliquots of each specific section ((i) cargo release experiments, Section 2.6; (ii) EOCs permeability across the intestinal barriers, Section 2.13; and (iii) in vivo pharmacokinetic assays, Section 2.17) was analyzed by HPLC. For Thy and Eug molecules, the mobile phase used was ACN:H_2_O 50:50 *v*/*v* with 0.05% of TFA and absorbance detector using λ_ex_ = 280 nm. For Cin, the mobile phase was ACN:H_2_O 85:15 *v*/*v* with 0.05% of TFA and λ_ex_ = 254 nm. 200 mL of volume injection were used and a flow of 1 mL/min was fixed. The methods were previously validated with adequate linearity, precision and accuracy (R > 0.99 and coefficient of variation < 5%).

### 2.8. Caco-2 Cell Culture Conditions

Human colon adenocarcinoma Caco-2 cells were acquired from the American Type Culture Collection (ATCC), and they were grown in DMEM medium supplemented with 10% FBS, 1% penicillin/streptomycin antibiotic and 1% nonessential aminoacids. Cells were maintained at 37 °C in incubator, with a humidified controlled atmosphere composed of 5% CO_2_ and 95% air and underwent passage when 80% confluence was reached.

### 2.9. MTT Cell Viability Assay

Caco-2 cell viability was determined by means of the MTT method. With this aim, Caco-2 cells were seeded in 96-well plates at a density of 2·10^4^ cells/well and they were incubated for 24 h. Then, all the tested M41-EOC-L particles were suspended in concentrations corresponding to payloads of 50, 100 and 200 μM. The M41-RhB-L system was used as control (control_part_), in particle concentrations of 250, 500 and 750 μg/mL (to match the maximum concentrations tested in each M41-EOC-L). Each particle-concentration was tested in eight wells and compared with cells absent of any MSP as negative control. Two independent experiments were performed to assess the assay-repeatability. After 24 h and 48 h of incubation, cells were washed with PBS in order to remove MSPs, and then 100 μL of MTT solution in non-supplemented DMEM (0.5 mg/mL) were added to each well. The plates were incubated for 2 h, after which the supernatant was removed and 100 μL of DMSO were poured into each well. The plate was softly shaken until complete solution of formazan crystals, and its absorbance was finally measured at λ_exc_ = 550 nm.

### 2.10. Caco-2 Cell Monolayer Culture

To obtain intestinal epithelium membranes, Caco-2 cells were seeded in six-well plates onto PET porous Millicell hanging cell culture inserts (Merck Millipore) (area 4.2 cm^2^; pore size 0.4 µm) in 2 mL of medium at a seeding density of 2.5 · 105 cells per insert in the apical side. Then, 3 mL of medium were added in the basolateral compartment. Culture medium was changed every 2–3 days and the seeded cells were grown for 21 days to allow cell differentiation into enterocyte-like cells, characteristic enzymes production and tight junctions formation [86]. Before starting all the experiments, transepithelial electrical resistance (TEER) of each insert was measured to confirm the correct formation of confluent intestinal monolayers.

### 2.11. Transepithelial Electrical Resistance (TEER) Measurements

The integrity of the monolayer formed by differentiated Caco-2 cells was evaluated by means of TEER measurements, before and after 24 h incubation for all the treatments, using a chop-stick electrode device (Millicell-ERS voltmeter, Millipore). TEER values were expressed as Ω·cm^2^ and were calculated according to the Equation (1):

TEER = [Ω _cell monolayer_ − Ω _insert (cell-free)_] × insert area (4.2 cm^2^) (1)

Inserts with TEER value > 600 Ω·cm^2^ were considered valid for conducting the corresponding experiments.

### 2.12. Intestinal Barriers Treatment with M41-#-L and Free EOCs

The Caco-2 cell epithelia were incubated in cell culture medium with each treatment, free EOCs (200 µM) and M41-#-L solids (concentration of particles corresponding to a 200 μM EOC-payload), during the time required to perform the assay: 90 min for EOCs permeability (see Section 2.13) and 24 h for the other interaction studies (Lucifer yellow, Section 2.14, and inflammatory response measurement, Section 2.15). In addition, some cell inserts were incubated with free medium at the same conditions as negative controls. TEER measurements were performed before and after each treatment to ascertain the validity of the membranes before the assay and to know the treatment’s effect on them.

### 2.13. EOCs Permeability across the Intestinal Barriers

Permeability assays were performed by using Caco-2 cell monolayers. EOCs-permeability across the membranes was performed in the apical-to-basolateral direction for the M41-EOC-L materials and the free EOCs. The transport experiments were performed in HBSS at 37 °C using an orbital shaker (50 rpm). The calculated concentration of particles corresponding to a 200 μM EOC-payload ((750 μg/mL for M41-Thy-L; 500 μg/mL for M41-Eug-L; 300 μg/mL for M41-Cin-L) and 200 μM free EOC) were pipetted in the apical (ap) side of the inserts in 2 mL of HBSS. Then, 100 μL samples were taken from the basolateral (bl) side of inserts after certain times of incubation (15, 30, 45 and 90 min), and the withdrawn volume was replaced with 100 μL of fresh HBSS buffer. EOC-concentrations of each sample were quantified by HPLC (see Section 2.7), and the accumulated amounts were plotted versus time (up to 90 min). The EOCs’ permeability from ap to bl (P_ab_) values were calculated as the slope of the obtained line.

### 2.14. Lucifer Yellow (LY) Assay

The integrity of the enterocyte-like-layer after 24 h of treatment with free EOCs and M41-#-L was evaluated by means of LY assay. This test measures the ability of this paracellular marker to cross the cell monolayer. After 24 h of incubation with the different treatments (M41-#-L, free-EOCs and non-treated controls), apical and basolateral media were collected and cell layers were washed twice with HBSS. Bl compartments were filled with HBSS, and the ap side with a LY solution in HBSS (0.4 mg/mL). Cell layers were incubated for 2 h at 37 °C, and then, 100 µL aliquots were taken from each insert at the bl side (including cell-free inserts). The aliquots were added into a black 96-well plate and their LY content was determined by fluorometric measurements (λ_ex_ = 428 nm, λ_ex_ = 536 nm). The transported LY percentage from-ap-to-bl side after treatment was compared to the transported LY in the control monolayers.

### 2.15. Inflammatory Response Measurement

Inflammatory cytokine and chemokine release (IL-8, MCP-1) was measured in the apical media from the enterocyte layers after 24 h of treatment with the corresponding M41-EOC-L and free EOCs. For the measurement, a Bio-Plex 1 MAGPIX TM Multiplex Reader (Bio-Rad) was employed according to the manufacturer-described-procedure. The cells were stimulated with 10 μg/mL LPS as positive control.

### 2.16. Cell Staining and Confocal Microscopy

For the observation of the microdevices’ interaction with cells, enterocyte-like layers were incubated for 24 h with M41-RhB-L and M41-EOC_C6_-L. After the incubation, cell monolayers were washed twice with PBS and fixed with 4% PAF for 30 min at room temperature. After washing, cells were permeabilized with 0.1% Triton ×100 for 5 min, and the process was stopped with blocking buffer solution (1% BSA in PBS) for 20 min. Cells were then stained with 0.1 nM Alexa Fluor 488 Phalloidin (M41-RhB-L treated cells) or Alexa Fluor 594 Phalloidin (M41-EOC_C6_-L treated cells) for 30 min to localize actin microfilaments. In order to localize cell nuclei, all the cells were stained with Hoechst 33,342 at a concentration of 5 µg/mL for 5 min. CLSM images were acquired using excitation wavelengths λ_ex_ = 405, 488, and 546 nm.

### 2.17. In Vivo Pharmacokinetic Assays

In vivo experiments with M41-Cin-L in a Wistar rat model were carried out in order to study the concentration of cinnamaldehyde, free and encapsulated, in plasma and in the different gastrointestinal sections. The animal study was approved by the Scientific Committee of the Faculty of Pharmacy Universidad Miguel Hernández, Elche (project reference DI-MBS-02-15) and followed the guidelines described in the EC Directive 86/609, the Council of the Europe Convention ETS 123 and Spanish national laws governing the use of animals in research (Real Decreto 223/1988, BOE 67, 18-3-98:8509-8511). To perform the in vivo assays, 12 male Wistar rats were previously fasted for 4 h. Subsequently, six animals were assigned to the trial group (g_T_), who were orally administered 150 mg of M41-Cin-L (containing 13.6 mg of Cin). The other six animals were assigned to the control group (g_C_), who were administered an equivalent dose of free Cin (13.6 mg). To carry out the experiments, both groups were organized into two batches with three subjects per batch (n = 3), one in which the assay was finished at 2 h (b_2h_), and another in which the digestive process was allowed to complete for up to 4 h (b_4h_). Plasma samples were collected at 30, 45, 60, 90 and 120 min from the b_2h_ batch of each group, and at 30, 45, 60, 90, 120, 180 and 240 min from the b_4h_ batch, and then the animals were sacrificed. The GIT of all the animals were excised, and divided into their corresponding physiological segment (stomach, duodenum, jejunum, ileum and colon). The tissues were washed with PBS, in a three-fold volume of the tissue’s weight, and finally homogenized using a glass homogenizer with Teflon pestle. Both blood samples and homogenized tissues were centrifuged (8000 rpm, 10 min), and deproteinized with cold methanol. The concentration of Cin in each sample was determined by HPLC following the methodology described in Section 2.7.

### 2.18. Statistical Analysis

Statistical results are showed as mean ± standard deviation. Mean values of two groups were analyzed using Student’s two tails t tests and differences between three or more groups were calculated with ANOVA as analysis of variance and Scheffé post-hoc test. A probability level of *p* < 0.05 was established as the significance criterion. The software used for the data analysis was SPSS v 22 (IBM United States) licensed by the Miguel Hernández University.

## 3. Results and Discussion

### 3.1. Design, Synthesis and Characterization of the Prepared Solids

**Scheme 1:**
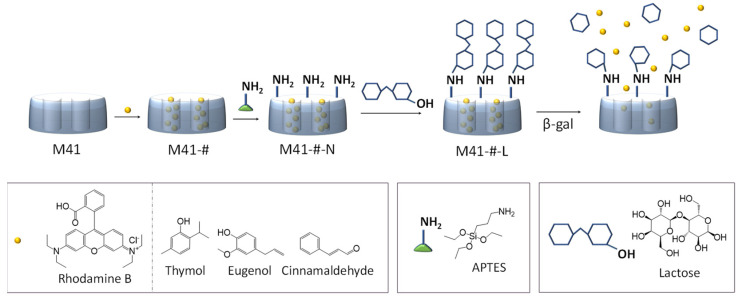
Representation of the M41-EOC-L synthesis process and subsequent cargo delivery performance under β-gal enzymatic action.

Mesoporous silica microparticles (M41) were prepared by the “atrane route” following previously described procedures [16]. A fluorophore (rhodamine B, RhB) and three EOCs (*Thy*, *Eug* and *Cin*) were loaded in the M41 microparticles (solids M41-#). M41-# supports were then reacted with APTES (solids M41-#-N) and lactose was attached to the solids by a condensation reaction between the saccharide’s anomeric carbon and the APTES-amino group to give the final capped solids (M41-#-N-L). As the pores are first loaded with RhB or EOCs the functionalization process with APTES and lactose is expected to occur mainly on the external surface of the M41 microparticles as shown in Scheme 1. In addition, EOCs-loaded lactose-capped materials with an additional hydrophilic fluorophore (coumarin 6, C6) were also prepared to carry out microscopy experiments.

**Figure 1:**
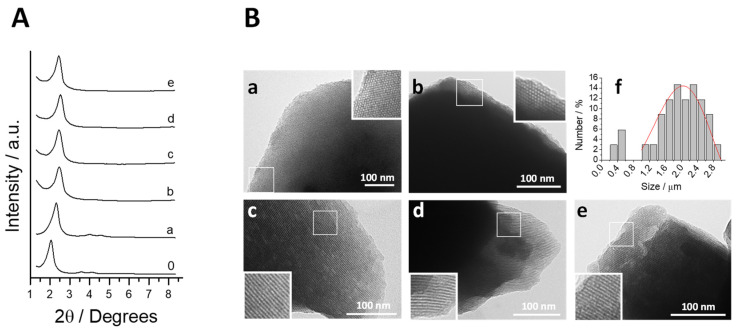
(**A**) Normalized powder X-ray patterns of all the synthesized solids. From bottom to top, the reported solids are the as made material (0), the calcined support M41 (**a**) and the loaded and functionalized final materials: M41-RhB-L (**b**), M41-Thy-L (**c**), M41-Eug-L (**d**) and M41-Cin-L (**e**). (**B**) TEM micrographs of starting bare material M41 (**a**), and loaded and functionalized solids M41-RhB-L (**b**), M41-Thy-L (**c**), M41-Eug-L (**d**) and M41-Cin-L (**e**), and particle size estimation (**f**). Insets correspond to a 2x magnification of the selected areas.

The synthesized solids were characterized by standard techniques. Normalized X-ray diffractograms of all the solids (MCM-41 “as made”, calcined particles (M41) and loaded-and-functionalized solids) are shown in Figure 1A. Four low-angle peaks can be observed in all the patterns, which are characteristic of a hexagonal channels-arrangement, and indexed from left to right as (1 0 0), (1 1 0), (2 0 0) and (2 1 0) Bragg reflections, respectively. Once the as made material was calcined, it can be observed that the (1 0 0) peak of the M41 particles is shifted to higher 2θ values compared with the as made one, phenomenon related with structure contraction due to the condensation of silanols in the calcination process. It also can be appreciated that in the curves corresponding to all the final loaded and functionalization solids, the reflections (1 1 0), (2 0 0) and (2 1 0) are vanished because of the high amount of organic matter which fills the pores. Although the increase in organic matter of the solids decreases the definition of their peaks, the clear presence of the peak (1 0 0) in all the diffractograms demonstrates that neither the calcination nor pore filling and functionalization processes modify the initial MCM-41 structure.

The mesoporous structure of the starting solid M41, and its persistence in the final solids after the loading and functionalization steps, was also confirmed through TEM images (Figure 1B). In these images, the typical matrix of the MCM-41 particles could be appreciated and visualized as alternating white and black stripes corresponding to the hexagonally arranged channels characteristics of the material’s structure. The hexagonal stacking was visible when the pores were frontally seen in the image. TEM images were also used to estimate the particles’ size and an average of 2.2 ± 0.8 µm (Figure 1B(f)) was obtained in the majority population.

**Figure 2:**
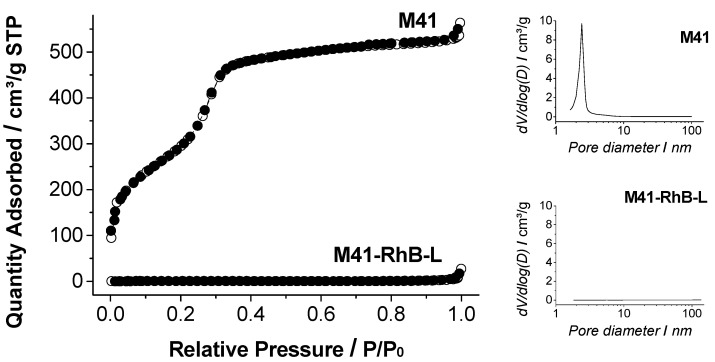
Left: Nitrogen adsorption (○) desorption (●) isotherms of the calcined starting material M41 and M41-RhB-L. Right: pore distribution graphs of M41 (**top**) and M41-RhB-L (**bottom**).

The N_2_ adsorption-desorption isotherm of the calcined support (M41) is shown in Figure 2. A typical curve of a mesoporous solid consisting of an adsorption step at P/P_0_ values between 0.1–0.3 is observed. The curve fits with a type IV isotherm, in which the increase produced in the gas absorption corresponds to a condensation of the N_2_ molecules within the mesopores of the inorganic structure [87]. The absence of hysteresis loop in this interval (0.1 < P/P_0_ < 0.3) and the narrow pore diameter distribution suggest the existence of cylindrical mesopores with a uniform structure, in which the processes of gas adsorption and desorption are developed following the same mechanism. The N_2_ adsorption-desorption isotherm of M41-RhB-L solid is typical of mesoporous systems with practically filled mesopores (Figure 2). The high amount of organic matter in M41-RhB-L avoids the adsorption of gas molecules, and consequently, the registered curve is completely flat. Accordingly, an absence of appreciable mesoporosity is observed and relatively low N_2_ adsorbed quantity and surface area values were obtained (see Table 1). Solids containing EOCs loaded into the pore voids could not be analyzed by this technique due to the EOCs volatility that would clog the porosimeter during the degassing pretreatment of the samples.

**Figure 3:**
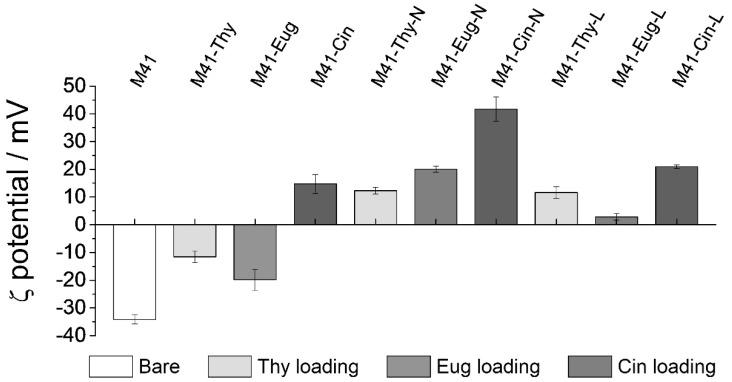
ζ potential values for solids in the different synthesis-stages. From left to right, bare material (M41), loaded supports (M41-EOC), APTES-functionalized materials (M41-EOC-N) and final gated microdevices (M41-EOC-L).

The different stages of the synthesis process, from loading to final functionalization with lactose through prior functionalization with APTES, were followed by ζ potential measurements. ζ potential (Figure 3) of the starting M41 solid is negative due to the presence of silanolate groups. This negative value is reduced for the loaded materials M41-Thy and M41-Eug. Moreover, a positive ζ potential for M41-Cin is observed, possibly due to the formation of some hemiacetal groups between the aldehyde group present in the *Cin* molecule and the silanolate groups in the M41-surface present at neutral pH, which compensates their initial negative charge. The alcohol which forms the active groups of *Thy* and *Eug* does not react with the M41 surface, ergo no important modifications of the surface’s charge is observed. Following with the next functionalization step, it can be appreciated how the APTES-moiety makes the external charge of the particles more positive due to the primary amines. Functionalization with lactose results in final solids still with a positive ζ potential.

The contents of organic matter of all the loaded and functionalized M41-EOC-L solids were determined by TGA, and they are listed in Table 2 as function of the remaining SiO_2_ residue (μg_OM_/mg SiO_2_). In addition, in order to compare the maximum payload of EOC Solidreleased from M41-EOC-L (see Section 3.3) with the loaded amount of EOC, the amount of EOC released per mg of solid (μg_EOC_/mg M41-EOC-L) are included in Table 2.

### 3.2. Biofluid Digestion: Release Profile and Structure-Integrity

**Figure 4:**
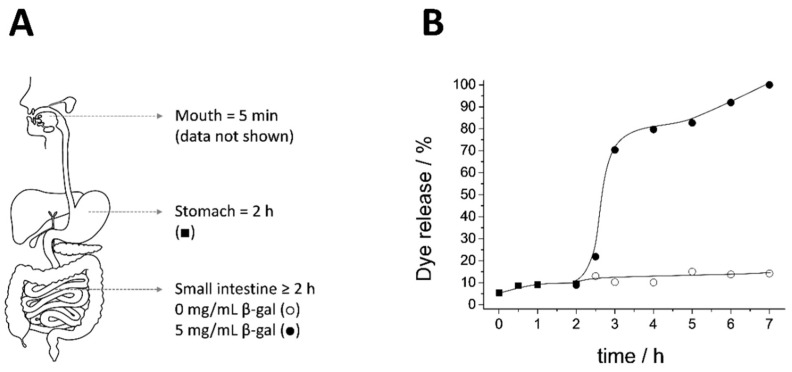
(**A**) Biofluid digestion scheme and retention times. (**B**) Biofluid digestion release profiles from M41-RhB-L in simulated gastric (■), and intestinal fluids in the absence (○) or in the presence of β-gal at a concentration of 5 mg/mL (●).

As previously stated, the performance of *Lac* moiety as gatekeeper triggered by the enzymatic action of β-gal was reported previously by some of us [79]. Based on this knowledge, this work aims to validate the performance of the *Lac* molecular gate in biological conditions. To achieve this goal, an biofluid digestion assay simulating GIT conditions [85] with the M41-RhB-L model material was accomplished. This procedure not only simulates the GIT by reproducing the oral, gastric, and SI environment (Figure 4A), but also considers all the important parameters during digestion process, such as pH changes, salinity, the most relevant enzymes or the residence times (Section 2.5). The obtained cargo release profiles from M41-RhB-L in the different GIT fluids are shown in Figure 4B. The procedure started with a stay of solid M41-RhB-L in the simulated saliva for 5 min (data not shown) followed by the gastric digestion step, where the gastric fluid was added. M41-RhB-L remained for 2 h in the stomach fluid, during which no release of RhB was observed. Finally, two different routes were followed to obtain the intestinal release profiles: a first route with addition of the β-gal enzyme (5 mg/mL) in the SI simulated fluid, and a second one without β-gal addition. While the first process showed a marked cargo release to the digestive fluid, for the latter no dye release was observed. The RhB release behavior of M41-RhB-L is similar to that reported in the literature under simplified conditions (water suspensions in the absence and in the presence of added β-gal) [79], thus demonstrating that the gating mechanism is equally efficient under simulated GIT conditions.

**Figure 5:**
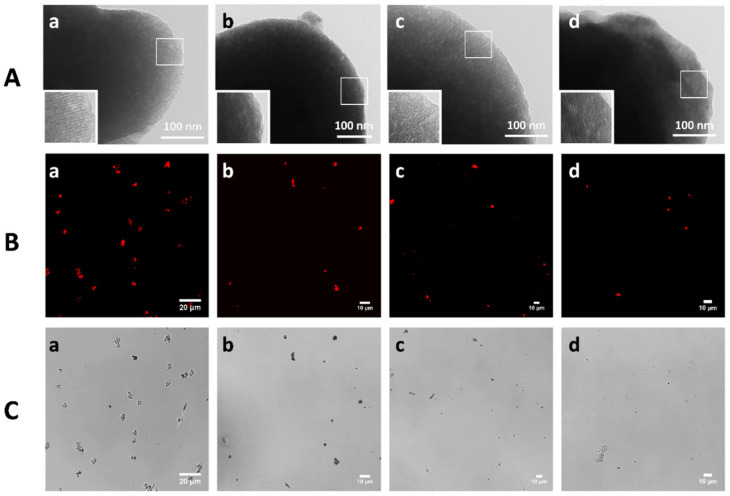
TEM (**A**), CLSM (**B**) and CLSM transmitted light (**C**) micrographs of M41-RhB-L suspended in water (**a**), and in the different simulated saliva (**b**), gastric fluid (**c**) and intestinal fluid (**d**), showing no degradation of the structured-material. TEM micrographs (**A**) were acquired after collecting, washing and drying the particles several times.

To study the possible degradation of the mesoporous support due to the aggressive environment of the digestive fluids (regardless of the triggering stimulus), the M41-RhB-L particles were studied using TEM and CLSM after each gastrointestinal step, and the obtained images are shown in Figure 5. The CLSM images were taken directly from the digestion fluids, while the samples for the TEM analysis were previously collected, washed and dried (for further details, see Section 2.5 for TEM and Section 2.15 for CLSM). TEM micrographs (Figure 5A) showed no significant changes in the M41 characteristic structure during the biofluid digestion. The typical striped structure was preserved from the initial material suspended in water (a) to the particles in the presence of saliva (b), gastric fluid (c) and finally all digestive fluids in the intestinal step (d), including duodenal fluid and bile salts. A loss of contrast was observed in the final micrograph (Figure 5A(d)), possibly due to the high amount of organic matter involved in this assay.

To support the results shown in the dye release graph (Figure 4B), aliquots of the different biofluid digestion steps were also subjected to CLSM (see Figure 5B,C). In these figures, the fluorescence of RhB inside the M41-RhB-L particles can be observed, both in particles suspended in water (Figure 5B(a)) and during all the digestion steps (Figure 5B(b–d)). Furthermore, complementary transmitted light of CLSM images (Figure 5C) confirmed that the cited fluorescence co-located with the particles. This result is in agreement with the release profile shown in Figure 4B, and together demonstrates the cargo-confinement into the M41 pores in absence of β-gal and the structure conservation after the biofluid digestion process. This result is in line with previous studies that demonstrated the stability of functionalized micro-sized M41 materials during biofluid digestion assays [88].

### 3.3. Cargo Release Experiments

**Figure 6:**
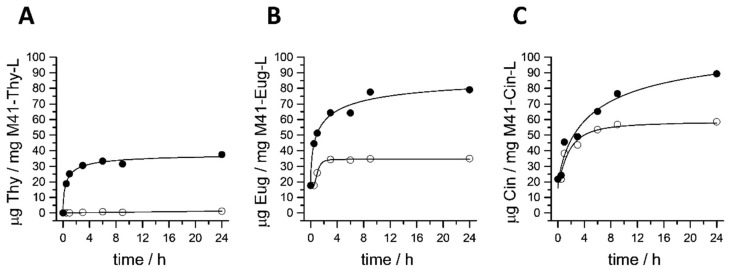
(**A**) *Thy*, (**B**) *Eug* and (**C**) *Cin* release profiles from their respective M41-EOC-L solids at 37 °C, in PBS in the absence (○) and in the presence of 5 mg/mL of β-gal (●) in PBS.

Once the in vitro control releasing ability of the gated microparticles under GIT simulated conditions was demonstrated, cargo-delivery assays from M41-EOC-L were carried out in the presence and in the absence of β-gal (5 mg/mL) in PBS at 37 °C. The cargo-delivery profiles were determined by HPLC and are shown in Figure 6.

M41-Thy-L shows a zero release in the absence of β-gal and reaches a delivery of 37.5 μg/mg of solid in the presence of the enzyme. The amount of *Thy* released is lower than that of M41-Eug-L and M41-Cin-L, yet it is also the EOC that is loaded in a lower amount (see Table 2). In comparison, the M41-Eug-L system (Figure 6B) presents a higher payload release than *Thy* (79.0 μg/mg solid at 24 h in the presence of β-gal) although in this case the lactose molecular gate is not able to completely inhibit cargo release in the absence of the enzyme. A similar situation is observed for M41-Cin-L. Cargo release in the absence of β-gal is of 58 μg/mg of solid, whereas this value increases to 89 μg/mg of solid with the enzyme. Moving to the payload’s delivery rate, both *Thy* and *Eug* molecules outflow from their respective M41-EOC-L solids more abruptly than *Cin*. In fact, after 3 h both M41-Thy-L and M41-Eug-L released ca. 81% of the maximum-released-payload in the presence of β-gal, while this value was only 55% for M41-Cin-L. This fact could be explained by the formation of a hemiacetal group between a fraction of the *Cin* molecules and the silanolates present in the M41-walls, which also affects to the ζ potential of the M41-Cin-L support (see Section 3.1). Even with the differences present in the M41-EOC-L solids, from their release profiles it can be concluded that *Lac* molecule was able to act as molecular gate and modulate the cargo delivery in the presence of β-gal.

### 3.4. In Vitro Interaction Studies between Caco-2 Cells and M41-#-L Materials

With the aim of investigating the interaction between the M41-#-L materials with the tissues present in the GIT, two different studies involving Caco-2 cells were performed: in the first one we used a simple model of undifferentiated Caco-2 cells and in the second an intestinal barrier model established from Caco-2 cells differentiation into enterocyte-like monolayers.

**Figure 7:**
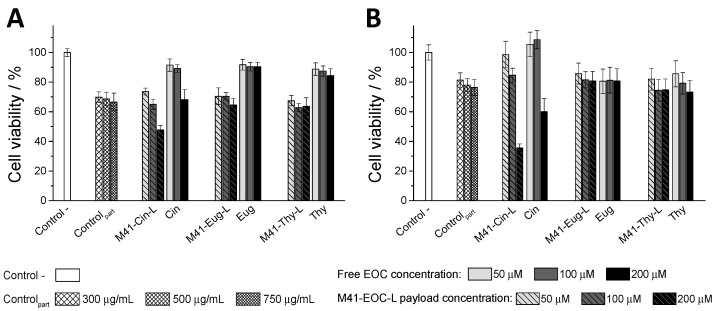
Cell viability of Caco-2 cells treated with the free EOCs and M41-EOC-L for (**A**) 24 h and (**B**) 48 h. Cell viability of free-EOCs treated cells (solid bars) and M41-EOC-L treated cells (striped bars) was compared with non-treated cells as negative control (empty white bars) and cells treated with the M41-RhB-L (striped white bars). Positive control (M41-RhB-L) bars have increasing density-mesh for increasing particle-concentration: 300, 500 and 750 μg/mL. EOC concentration, both as free compounds or as the microdevices’ released payload, was increased from 50 μM (light gray bars) to 100 μM (dark gray bars) and finally 200 μM (black bars). To achieve this payload concentration, the tested particle concentration of the different M41-EOC-L microdevices was: 75, 150 and 300 μg/mL for M41-Cin-L; 125, 250 and 500 μg/mL for M41-Eug-L; and 190, 375 and 750 μg/mL for M41-Thy-L. Data correspond to the average of two different experiments (8-well per experiment), and error bars represent their standard deviation.

The viability of Caco-2 cells treated with the M41-#-L systems and free EOCs was determined by using the MTT method. The conversion of MTT (yellow-colored compound) into formazan (purple-colored compound) is directly proportional to the number of living cells, and can be spectroscopically quantified. EOCs antioxidant capacity has been reported to result in anti-inflammatory properties at low concentrations, [89,90,91] and with anti-cancer effects at high concentrations [57,58,59,92,93]. Accordingly, EOC concentrations in the cell assays were chosen from data reported in the literature [57,58,59,89,90,91,92] and were fixed at 50, 100 and 200 μM to evaluate the effect of a wide range of concentrations on Caco-2 cells. M41-EOC-L particles were added to release final equivalent concentrations of the loaded EOCs (i.e., 75, 150 and 300 μg/mL for M41-Cin-L; 125, 250 and 500 μg/mL for M41-Eug-L; 190, 375 and 750 μg/mL for M41-Thy-L). Non-treated cells were used as negative control and M41-RhB-L (at concentrations of 300, 500, and 750 μg/mL) was tested to check the support-toxicity per se (control_part_). Caco-2 cells were exposed to free EOCs and M41-#-L for 24 and 48 h, and the obtained results are shown in Figure 7.

Despite the exposure to critical particle concentrations such as 750 μg/mL, the high cell viability obtained for Caco-2 cells treated with M41-RhB-L (Figure 7, positive control) indicates that the lactose-functionalized M41 solid is not toxic. The slight viability-reduction that can be observed when compared with control cells could be related with cell coating due to particle sedimentation, which can increase the cellular uptake and consequently, the measured cytotoxicity [72,94]. Cell viability after 24 h upon treatment (Figure 7A) shows that free EOCs at the tested concentrations are not toxic. Only *Cin* at the highest concentration (200 μM) slightly reduces cell-viability. Toxicity assays with M41-EOC-L are similar to those found for M41-RhB-L except for M41-Cin-L at high concentration that significantly reduced cell viability to values as low as ca. 30% at 48 h.

### 3.5. In Vitro Interaction Studies between Intestinal Barriers and M41-#-L

As stated before, one of the most important objectives of this work was to study the interaction of the designed microdevices with the GIT (both with in vitro and in vivo models), especially with the intestinal epithelium, since it is one of the most important cellular barriers in the mammalian organism. This barrier comprises various vitally important functions, from the nutrient uptake to the protection against pathogenic organisms, including the shelter of the intestinal microbiota [95]. Homeostasis of the intestinal barrier is compromised by numerous diseases of the digestive system, such as inflammatory bowel diseases or some types of cancer [96,97,98,99,100,101]. Alterations in the intestinal barrier, whether profitable to induce treatment or damaging due to pathologies or drug side effects, end up affecting the entire organism [101]. Therefore, the study of the interaction of drug carriers with an intestinal barrier model, either in vitro or in vivo, is essential to validate their oral administration. The in vitro model of intestinal epithelium generated by Caco-2 cells differentiated into enterocyte-like cells is a versatile tool for these studies [102]. It is a low-cost assay without ethical impact, which allows evaluating the effectiveness of systems in an early stage of development. Furthermore, and as a key fact for the performance of the particles studied here, the intestinal barrier model is capable of producing β-gal with an activity similar to that of the human intestine [34,77,80].

**Scheme 2:**
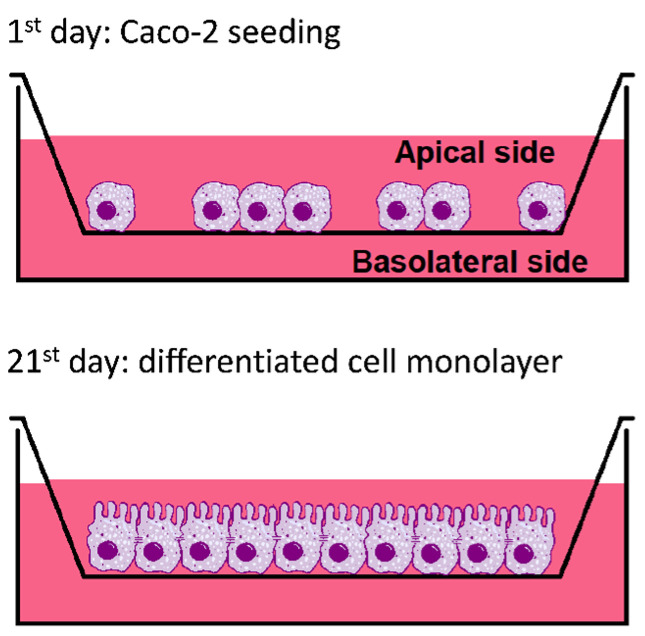
Representation of the Caco-2 seeding and differentiation after 21 days to intestinal barrier in vitro model.

This in vitro model of intestinal barrier was obtained establishing a cell layer by seeding and growing human intestinal epithelial cells for 21 days onto porous inserts [77]. The procedure is detailed in Section 2.10 and outlined in Scheme 2. Transepithelial electrical resistance (TEER) measurements were performed before each assay (see Section 2.11 for details), and they verified the complete membrane formation to ensure the development of its characteristic structures, such as microvilli and tight junctions between cells. Intestinal barriers were exposed to the maximum particle concentrations necessary to release an EOC payload of 200 μM, as it was done with undifferentiated Caco-2 cells (Section 3.4, *vide supra*), to perform the assays described below.

#### 3.5.1. EOCs Permeability through the Intestinal Barriers

**Figure 8:**
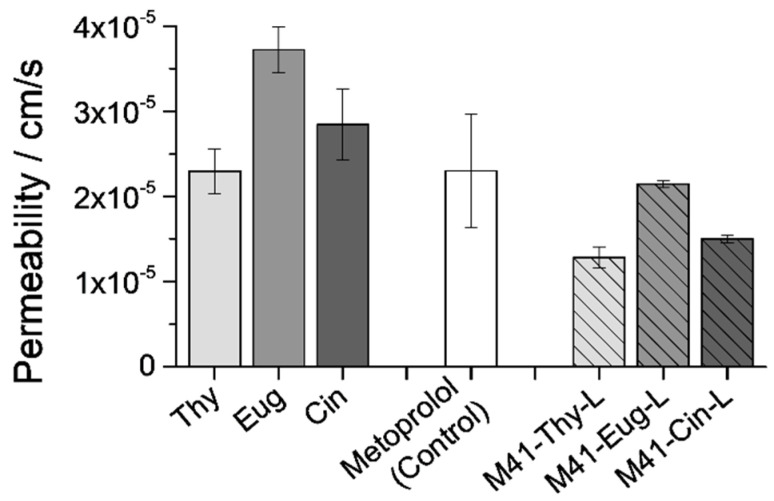
Apical to basolateral permeability (P_ab_) values obtained from the intestinal membrane treatment with the three different EOCs, both free (solid bars) or encapsulated into the M41-EOC-L microdevices (striped bars). Metoprolol’s permeability values were also added as standard permeability reference (white bar). Provided data represent the average of three different experiments measured in triplicate, and the error bars represent the standard deviation.

The permeability assay measures the speed of passage of a compound through the intestinal membrane, from the apical (*ap*) compartment to the basolateral (*bl*) side, during the assay time (90 min). Apical to basolateral permeability (P_ab_) values of EOCs were determined using the Caco-2 monolayers as an in vitro model able to predict human absorption, simulating the passage of compounds from the intestinal lumen (*ap*) to the plasma (*bl*) across the intestinal membrane [94,102]. To perform the assay, EOCs (free or encapsulated into M41-EOC-L microdevices) were placed in the *ap* side, and during the 90 min of incubation, different samples were taken from the *bl* side. Calculated P_ab_ values of the different EOCs, both free and encapsulated into the M41-EOC-L microdevices, are shown in Figure 8, where also the P_ab_ value of Metoprolol has been included as permeability reference standard.

Statistical analysis was performed and Student’s two tails t tests showed statistically significant differences (*p* < 0.001) between EOCs’ permeability (P_ab_) values obtained after the administration of free solution, in comparison with encapsulated drugs. Levene’s test of homogeneity of variance for the design demonstrated the homogeneity between values obtained in replicate experiments.

The depicted data in Figure 8 show high P_ab_ values for the free EOCs compared to the reference standard, thus being able to define them as high permeability compounds. These results are consistent with the lipophilic nature of these EOCs [62,63], which causes them to rapidly diffuse through the epithelial membrane. However, when EOCs were encapsulated into the M41-EOC-L microdevices, their P_ab_ values become lower for the three EOCs tested, even lower than the standard reference. This result indicates that the EOCs loading and protection in the designed system slows down the payload’s release transport across the monolayer. This reduction of P_ab_ values is especially interesting as it would mean that the designed carriers could produce *(i)* the prevention of drug access to systemic circulation, *(ii)* the improvement of the therapeutic efficacy in the colon, *(iii)* the reduction of systemic toxicity and *(iv)* the minimization of adverse side effects.

#### 3.5.2. Intestinal Membrane Treatment with Free EOCs and M41-#-L

**Figure 9:**
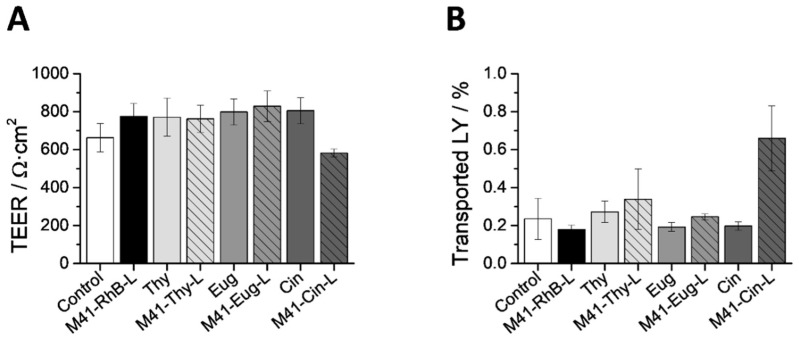
(**A**) TEER measurement values of non-treated intestinal barriers as negative control (white bar), treated with M41-RhB-L non-active microdevice as positive control (black bar) and intestinal barriers after 24 h of exposure to free EOCs (solid bars) or encapsulated into the M41-EOC-L microdevices (striped bars). (**B**) Transported LY (%) through the intestinal barrier after 24 h of exposure to free EOCs (solid bars) or encapsulated into the M41-EOC-L microdevices (striped bars) compared with non-treated membranes as negative control (white bar) or treated with M41-RhB-L (black bar). Data correspond to the average of three different experiments measured in triplicate, and error bars represent their standard deviation.

Once studied the effect of encapsulation on EOCs’ permeability, the next step to study the interaction of microdevices with the intestinal membranes was to evaluate the effect of their treatment during longer exposure times. The enterocyte-like membranes were exposed for 24 h to a critical particle concentration, necessary to release a payload’s concentration of 200 μM. The integrity of the membranes after treatment was determined by TEER (*Transepithelial electric resistance*) measurement and by the percentage of transported LY (*Lucifer yellow*) from *ap* to *bl* side (Figure 9A,B, respectively).

As it can be observed in the graphs, TEER values of monolayers treated with free-EOCs and treated with the M41-Thy-L and M41-Eug-L supports did not differ from those obtained from the negative control (untreated membranes, white bar) or the control_part_ (M41-RhB-L, black bar). However, the TEER values of the monolayers treated with M41-Cin-L were much lower than the control ones, which could be related with an alteration produced in the intestinal epithelium. Focusing on the transport of LY, a paracellular marker that manifests membrane-alterations, it can be seen that the values of all the treatments were similar to the control, with the same exception of the M41-Cin-L system. From the obtained data, it can be deduced that the M41-#-L system does not alter the intestinal-membranes-viability per se. Only the M41-Cin-L system causes a response in the epithelial membranes, which is consistent with the Caco-2 cell viability data obtained in Section 3.4 and also with the EOCs permeability study (Section 3.5.1, *vide supra*). The combination of the increased cytotoxicity for Caco-2 cells of *Cin* when it is encapsulated and its reduced permeability trough the membranes (increasing its residence time in the *ap* side of membranes) may explain both the TEER and LY results.

#### 3.5.3. Inflammatory Response of Intestinal Epithelium to M41-EOC-L and Free EOCs

It has been reported in the literature that an inflammation response of the intestinal epithelium can be triggered by its exposure to different materials, depending on their physicochemical characteristics [103,104,105]. Focusing on studies with silica particles, micro-sized silica particles have been proved to be less aggressive than nano-sized ones, and furthermore, the inflicted damaged is reduced when their surface was functionalized with organic moieties [103]. The present section aims to know the existence of an inflammatory reaction produced in the intestinal membranes exposed to free EOCs or M41-EOC-L. Hence, the secretion of two inflammatory cytokines, IL-8 and MCP-1, in the *ap* media was measured to evaluate the monolayer inflammatory response. IL-8 and MCP-1 were selected as monitored cytokines due to their frequent increase in both Caco-2 cultures subjected to inflammatory agents [106,107] and in patients with inflammatory diseases [108,109].

**Figure 10:**
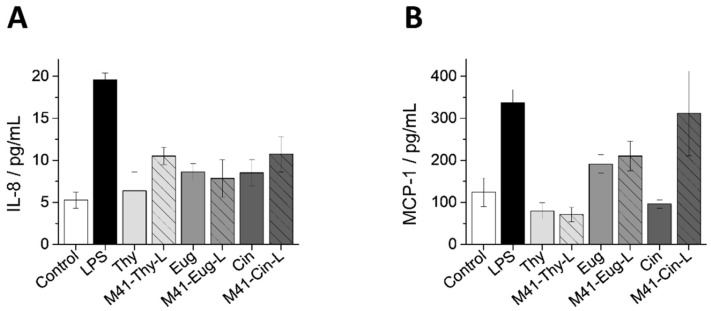
(**A**) IL-8 and (**B**) MCP-1 cytokine production in the apical compartment as inflammatory response of intestinal barriers exposed to the quoted treatments. Non-treated control intestinal barriers (white bar), treated with LPS as inflammatory control (black bar) and intestinal barriers after 24 h of exposure to free EOCs (solid bars) or released from M41-EOC-L (striped bars). Provided data represent the average of three different experiments measured in triplicate, and the error bars represent the standard deviation.

The cytokine production values of the treated-membranes with the different treatments in an EOC concentration of 200 μM (both free and in M41-EOC-L) for 24 h was measured. The obtained values are shown in Figure 10, which also shows data obtained from untreated-cells and cells treated with lipopolysaccharide (LPS) as negative and positive controls, respectively. The IL-8 levels of cells treated with free EOCs and M41-EOC-L (Figure 10A) are slightly higher than the negative control, but considerably lower than that measured in LPS-treated membranes. Furthermore, the M41-Cin-L support has the highest IL-8 production among all the performed treatments, closely followed by the M41-Thy-L. In relation with the MCP-1 cytokine it can be observed in Figure 10B that *Eug* treatment of the membranes increases its levels, both in the free and the encapsulated administrations. Moreover, in the case of the *Cin* the treatment with the M41-Cin-L produces very high values of MCP-1, comparable with the secretion of LPS-stimulated membranes, and quite higher than the treatment with free-*Cin*. From a general view, EOCs encapsulated in M41-EOC-L promote a greater inflammatory response than the corresponding free compounds. This is especially so for M41-Cin-L.

#### 3.5.4. CLSM Imaging of the Intestinal Barrier’s Interaction with M41-#-L

**Figure 11:**
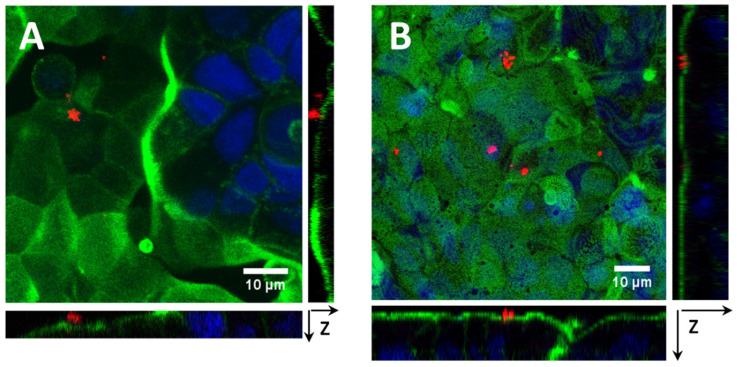
CLSM images of confluent intestinal barriers after 24 h of exposure to M41-RhB-L (**A**) and M41-Cin_C6_-L (**B**). Actin microfilaments (stained with Alexa Fluor Phalloidin)) in green, cell nuclei (stained with Hoechst 33342) in blue, and M41-#-L particles in red (see Section 2.16 for details). Lateral boxes represent z-stack projections along x–z and y–z axis.

In addition to the previous assays, the M41-#-L interaction with enterocyte-like membranes was also investigated by means of CLSM. Since M41-Cin-L particles at high concentrations produce membrane alterations, a low particle concentration (50 μg/mL) was selected to perform microscopy assays. Figure 11 shows CLSM micrographs of enterocyte membranes treated with M41-RhB-L (Figure 11A) and M41-Cin_C6_-L (Figure 11B), after 24 h. Microscopy assays were performed with the other two M41-EOC_C6_-L systems, but due to the results’ similarity and the images’ redundancy, data are not shown.

As it can be seen in Figure 11, few spots corresponding to the silica particles can be seen. The images confirm, qualitatively and from a morphological point of view, that the treatment with this particle concentration does not affect the membrane integrity. Moreover, CLSM z-sectioning of the intestinal membrane demonstrates the extracellular particle-localization. As expected from comparison with similar studies in the literature [110], there is a low particle internalization by the enterocyte membranes. As it has been reported in multiple studies, the particle uptake by the intestinal endothelium is reduced due to the existence of tight junctions between cells and a dense brush border of microvilli [102,110,111]. Therefore, the non-internalization of the microdevices by the intestinal membranes would favor their permanence in the intestinal lumen that jointly with the triggering action of the β-gal secreted by the microvilli may to extend the effect of the encapsulated EOC-payload even reaching the large intestine.

### 3.6. In Vivo Pharmacokinetic Assays

Due to the existing constraints of in vitro models, where certain parameters are difficult to simulate (e.g., exact enzymatic action, real composition of fluids and their pH, gastric emptying and digestive motility, exact mechanisms of internalization across membranes, etc.), we carried out an in vivo pharmacokinetic study with M41-Cin-L as *(i)* it exhibited the highest EOC-loading efficiency, *(ii)* it reduced Caco-2 cell viability to the lowest values and with the lowest particle concentration, *(iii)* it achieved the greater pro-inflammatory response in the enterocyte monolayers, necessary for a regenerative action, and *(iv)* the *Cin*-transport through the membrane was twice slowed when compared with free-*Cin*.

**Scheme 3:**
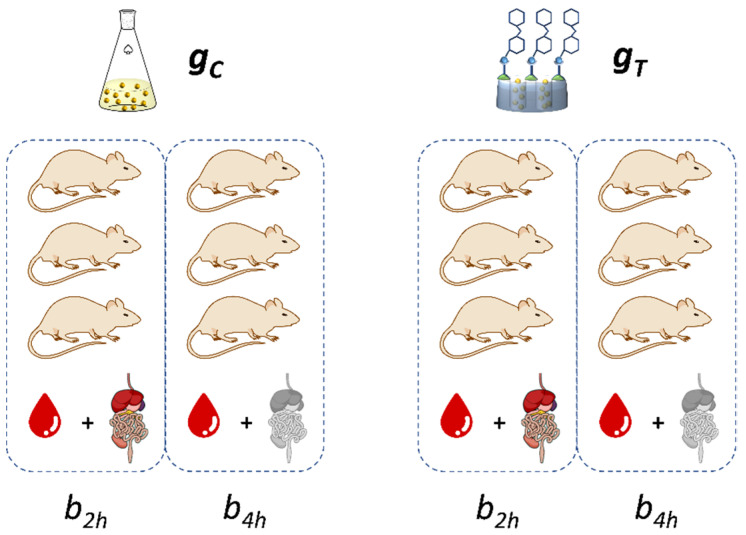
Representation of the in vivo experiment’s design. Animals were divided into two groups, one for free-*Cin* administration as control (*g_C_*) and a second one for M41-Cin-L administration as trial group (*g_T_*). Additionally, both groups were subdivided into two batches, in which digestion assay was stopped after 2 h (*b_2h_*), and a second group where the digestion was allowed to complete during 4 h (*b_4h_*). Plasma samples of all the subjects were considered, but only GIT sections of the b_2h_ group were considered, since they still maintain the treatments into the intestinal lumen (non-excreted).

To perform the pharmacokinetic studies, male Wistar rats were divided into two groups (n = 6 in each group). Subjects in control group (*g_C_*) were orally administered 2 mL of *Cin* solution (13.8 mg *Cin* in 2 mL PBS), and subjects from the second group (*g_T_*) were orally administered with 150 mg of M41-Cin-L (equivalent *Cin* payload of 13.8 mg) suspended in 2 mL of PBS. Additionally, both groups were organized into two batches (n = 3 in each group), one in which digestion assay was stopped after 2 h (*b_2h_*), and a second group in which the digestion was allowed to complete for 4 h (*b_4h_*). These two batches were established for the experiment since GIT samples were needed in short times where the animals had not excreted the treatments (2 h), but plasma samples were needed up to longer times (4 h) for the pharmacokinetic profile of *Cin*. After the treatments’ administration and subsequent plasma sampling, animals were euthanised (batch *b_2h_* at time = 2 h, and batch *b_4h_* at time = 4 h) and their GIT were excised to quantify the *Cin* concentration in the different GIT sections. The experiment’s design is outlined in Scheme 3, and although the GITs of subjects from *b_4h_* were also excised, only data from b_2h_ have been represented.

#### 3.6.1. Cin Quantification in Plasma samples: Pharmacokinetic Parameters

**Figure 12:**
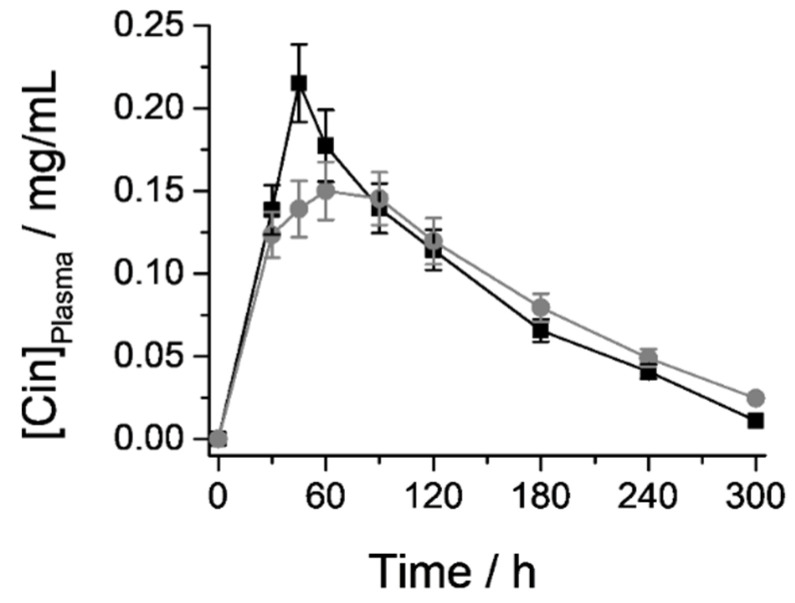
Cinnamaldehyde (*Cin*) concentrations in plasma and after oral administration of a dose of 13,6 mg *Cin* in the different formulations: free (black ■) or encapsulated into the M41-Cin-L microdevice (gray ●). The number of individues in the groups were n = 6 up to 120 min, and n = 3 up to 240 min. Provided data represent data average, and error bars represent the standard deviation.

The variation with time of the *Cin* concentration in plasma for both groups of animals is shown in Figure 12, and the calculated values of different pharmacokinetic parameters are listed in Table 3. From the data shown in Figure 12 and Table 3, it can be seen that subjects from *g_C_* group (animals treated with free *Cin*) showed maximum concentration of *Cin* molecule (C_max_) at 45 min after dose administration (Figure 12, black line). However, the C_max_ value of subjects from *g_T_* group (animals treated with M41-Cin-L) appears at 60 min, slightly more delayed (Figure 12, gray line). This difference can be related with the decrease in *Cin* permeability thanks to its protection into the hybrid system M41-Cin-L. As already mentioned above, and demonstrated with the results in Section 3.5.1, *Cin* is a lipophilic molecule with high permeability whose absorption is produced almost immediately after gastric emptying. However, compound’s protection inside the M41-Cin-L system delays its release until the action of intestinal β-gal, which is also consistent with the in vitro permeability results described in Section 3.5.1. Moreover, C_max_ obtained in the plasma samples for the M41-Cin-L system was 20% lower than the peak obtained for the free compound (0.22 and 0.15 mg/mL respectively), which in a possible co-administration of several drugs reduces the potential risk of adverse effects derived from drugs interaction.

The other two pharmacokinetic parameters are: the area under the curve (AUC), which is related to the total amount of compound in plasma, and the time at which half of cited compound has been metabolized (t_½_). Based on these parameters, it can be seen that the amount of compound in the plasma is practically the same (almost identical AUC values, 31.02 and 30.76 for free *Cin* and M41-Cin-L, respectively), although the incorporation is faster for the free compound (lower t_½_ value, 81.55 min compared with 93.67 min for M41-Cin-L). This again corroborates the decrease of the *Cin*-permeability when it is encapsulated inM41-Cin-L.

#### 3.6.2. Cin Quantification in the GIT Lumen

**Figure 13:**
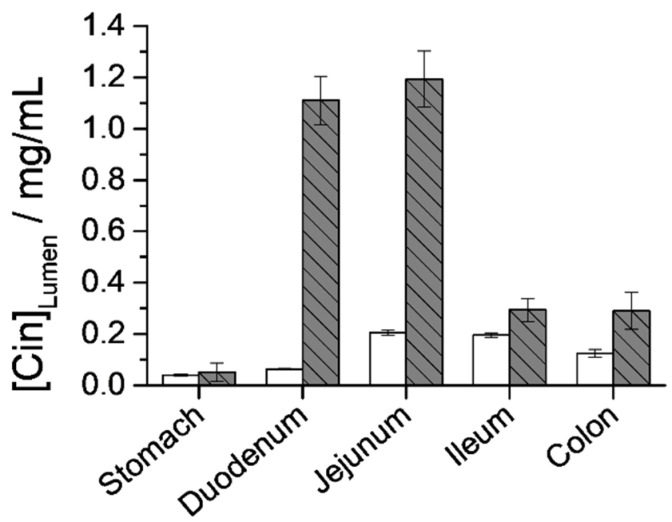
Cinnamaldehyde (*Cin*) concentrations in the detailed GIT sections after 2 h from an oral administration of a dose of 13,6 mg *Cin* in the different formulations: free (white bars) or encapsulated into the M41-Cin-L microdevice (gray stripped bars). The subjects’ number was n = 3, provided data represent the data average, and error bars represent the standard deviation.

Finally, *Cin* concentration in the different intestine sections from the excised GIT was quantified. After 4 h of oral administration *Cin* levels in all the GIT sections (*b_4h_*) were undetectable, both for the free-Cin and M41-EOC-L (data not shown). On the other hand, the obtained results after 2 h of digestion (*b_2h_*) (Figure 13) show that although *Cin* levels are also negligible in almost all the GIT-sections when the compound was administered free, it’s much higher when the treatment administration is performed with the M41-Cin-L system: until 20-fold higher in duodenum, 6-fold higher in jejunum, and 2-fold higher in ileum and colon.

Statistical analysis was performed and Scheffe tests were selected to compare Cinnamaldehyde (Cin) concentrations obtained in the different regional intestinal drug accumulation after 2 h from an oral administration. Homogeneity of variance was demonstrated by Levene’s test and statistics differences were describing by Scheffe post-hoc test between Cin-presence in all physiologic segmental parts of intestine. Cin concentration was different in all segments and when comparison between free solution or encapsulated drug was performed.

This distribution profile in the different sections of the GIT shows that the M41-Cin-L system is capable of prolonging the *Cin*-presence along the intestinal lumen compared to the administration of the free compound. This fact benefits the administration of *Cin* as potential antitumor drug to treat intestinal tract tumors in a more effective way, since the greater permanence in the intestinal lumen and the progressive action of the β-gal secreted along the SI would maintain the *Cin*-bioavailability. Furthermore, this microdevice may be suitable for other types of drugs intended to exert their effects in the intestine, such as other antitumor drugs, antiparasitic, anti-inflammatories, immunosuppressant, corticosteroids or any other bioactive compound whose target is the gut itself.

## 4. Conclusions

In this work, the ability of lactose of capping mesoporous silica particles (MSPs) loaded with essential oil components (EOCs) for controlled delivery specifically in small intestine conditions was investigated. The effects of the digestive process on the lactose-capped systems were studied using a biofluid digestion assay, after which no structural changes in the essayed solids were observed. Then, the interaction of lactose-gated systems with different in vitro and in vivo intestinal models was studied. Interaction with Caco-2 cells resulted in a greater cell-viability-reduction by the cinnamaldehyde-loaded microdevice (M41-Cin-L) compared with the free compound. Interaction with an intestinal membrane model stablished from Caco-2 differentiation into enterocyte-like monolayers, demonstrated a Cin permeability decrease across the membrane by means of its encapsulation in M41-Cin-L. This permeability-reduction involves a progressive action of the M41-Cin-L system, in which a more sustained payload bioavailability is achieved compared with the obtained with the free EOC administration. Moreover, a pro-inflammatory effect of M41-Cin-L on the intestinal membranes, that could be a needed cell response to promote a curative action, was observed. Finally, the effect of M41-Cin-L was studied in an in vivo model of the Wistar rat. Through these assays, the permeability reduction observed in vitro was confirmed. A reduction in cinnamaldehyde plasma levels was observed. Moreover, the distribution profile shows that the M41-Cin-L system is capable of prolonging the Cin-presence along the intestinal lumen compared to the administration of the free compound. This effect, together with the reported anti-cancer properties of Cin, makes the M41-Cin-L microdevice a system with potential use to treat tumors in distant sections of the small intestine and even the colon. The obtained results suggest that the M41-payload-L could be a potential hybrid microdevice for the protection and administration of other bioactive molecules whose action place is the small intestine and colon, such as anti-inflammatory, antiparasitic or other antitumor drugs.

## Figures and Tables

**Table 1:** BET specific surface values, pore volumes and pore sizes calculated from the N_2_ adsorption-desorption isotherms for bare and final-model solids.

Solid	S_BET_ (m^2^/g)	Pore Volume ^a^ (cm^3^/g)	Pore Size ^a,b^ (nm)
M41	1098.3	1.0	2.5
M41-RhB-L	2.6	0.0	-

^a^ Pore volumes and pore sizes are restricted to intraparticle mesopores. ^b^ Pore size was estimated by using the BJH model applied on the adsorption branch of the isotherm.

**Table 2:** Organic matter content (μg_OM_/mg SiO_2_) and EOC payload released (μg/mg of M41-EOC-L) for each M41-EOC-L microdevice.

Solid	μg_OM_/mg SiO_2_	μg_EOC_/mg M41-EOC-L Released
M41-Thy-L	311	37.5 ^a^
M41-Eug-L	446	79.0 ^a^
M41-Cin-L	758	89.3 ^a^

^a^ Maximum payload released (μg_EOC_/mg M41-EOC-L) from data in Section 3.3.

**Table 3:** Pharmacokinetic parameters calculated from plasma samples after oral administration of a Cin dose of 13.6 mg both free and encapsulated into M41-Cin-L microdevice.

Parameter	*Cin* Free	M41-Cin-L	% Relative (M41-Cin-L/Cin Free)
t_max_	45 min	60 min	↑ 33.3%
C_max_	0.22 mg/mL	0.15 mg/mL	↓ 31.8%
AUC	31.02	30.76 mg·min/mL	↓ 0.8%
t_½_	81.55 min	93.67 min	↑ 14.9%

## Data Availability

Not applicable.
